# DELirium Prediction Based on Hospital Information (Delphi) in General Surgery Patients

**DOI:** 10.1097/MD.0000000000003072

**Published:** 2016-03-25

**Authors:** Min Young Kim, Ui Jun Park, Hyoung Tae Kim, Won Hyun Cho

**Affiliations:** From the Department of Nursing (MYK), Ulsan University, Ulsan, Korea; and Department of Surgery (UJP, HTK, WHC), Dongsan Medical Center, Keimyung University, Daegu, Korea.

## Abstract

To develop a simple and accurate delirium prediction score that would allow identification of individuals with a high probability of postoperative delirium on the basis of preoperative and immediate postoperative data.

Postoperative delirium, although transient, is associated with adverse outcomes after surgery. However, there has been no appropriate tool to predict postoperative delirium.

This was a prospective observational single-center study, which consisted of the development of the DELirium Prediction based on Hospital Information (Delphi) score (n = 561) and its validation (n = 533). We collected potential risk factors for postoperative delirium, which were identified by conducting a comprehensive review of the literatures.

Age, low physical activity, hearing impairment, heavy alcoholism, history of prior delirium, intensive care unit (ICU) admission, emergency surgery, open surgery, and increased preoperative C-reactive protein were identified as independent predictors of postoperative delirium. The Delphi score was generated using logistic regression coefficients. The maximum Delphi score was 15 and the optimal cut-off point identified with the Youden index was 6.5. Generated area under the (AUC) of the receiver operating characteristic (ROC) curve was 0.911 (95% CI: 0.88–0.94). In the validation study, the calculated AUC of the ROC curve based on the Delphi score was 0.938 (95% Cl: 0.91–0.97). We divided the validation cohort into the low-risk group (Delphi score 0–6) and high-risk group (7–15). Sensitivity of Delphi score was 80.8% and specificity 92.5%.

Our proposed Delphi score could help health-care provider to predict the development of delirium and make possible targeted intervention to prevent delirium in high-risk surgery patients.

## INTRODUCTION

Delirium is a clinical syndrome defined as a disturbance of consciousness and cognition over a short period of time, which has a fluctuating course.^1^ The incidence of delirium after general surgery ranges between 10% and 50% depending on study populations and institutions.^2–5^ Age has been reported to be an independent marker for the development of postoperative delirium; in older patients, delirium leads to numerous detrimental effects.^6,7^ The South Korean population is aging rapidly and the number of surgeries performed per year rose from more than 266,000 in 2005 to more than 788,000 in 2013—an increase of 200%—and this trend is expected to continue over the next decades.^8^ As older patients are admitted for surgery, the incidence of postoperative delirium is also expected to increase.

Postoperative delirium, although transient, is associated with adverse outcomes, which range from a minor functional decline to postoperative death in the hospital and consequently increased health care costs.^9,10^ It has been suggested that the correction of modifiable risk factors is effective in delirium prevention and delirium prevention programs can succeed in decreasing the duration of delirium.^11,12^ However, general prevention for all surgical patients is not cost-effective. Thus, a predictive model for delirium is necessary to identify high-risk patients for monitoring and proactive implementation of preventive strategies.

Although several scoring systems for predicting delirium have been developed and used, they have some limitations for their application in general surgery practice. Because some systems have been developed for medical patients, they are not appropriate to predict postoperative delirium in surgical patients.^13–15^ In addition, the use of all previously developed delirium prediction scoring systems is complicated because they incorporate other scoring systems.^13,14,16,17^

The aim of this study was to develop a simple and accurate delirium prediction score that would allow identification of the individuals with a high probability of postoperative delirium on the basis of preoperative and immediate postoperative data.

## METHODS

### Design

This was a prospective observational single-center study, which consisted of two parts: the development of the DELirium Prediction based on Hospital Information (Delphi) score using data from patients who underwent major general surgery and validation of the developed Delphi score in a different prospective cohort in the same hospital.

### Identification of Potential Risk Factors

**FIGURE 1:**
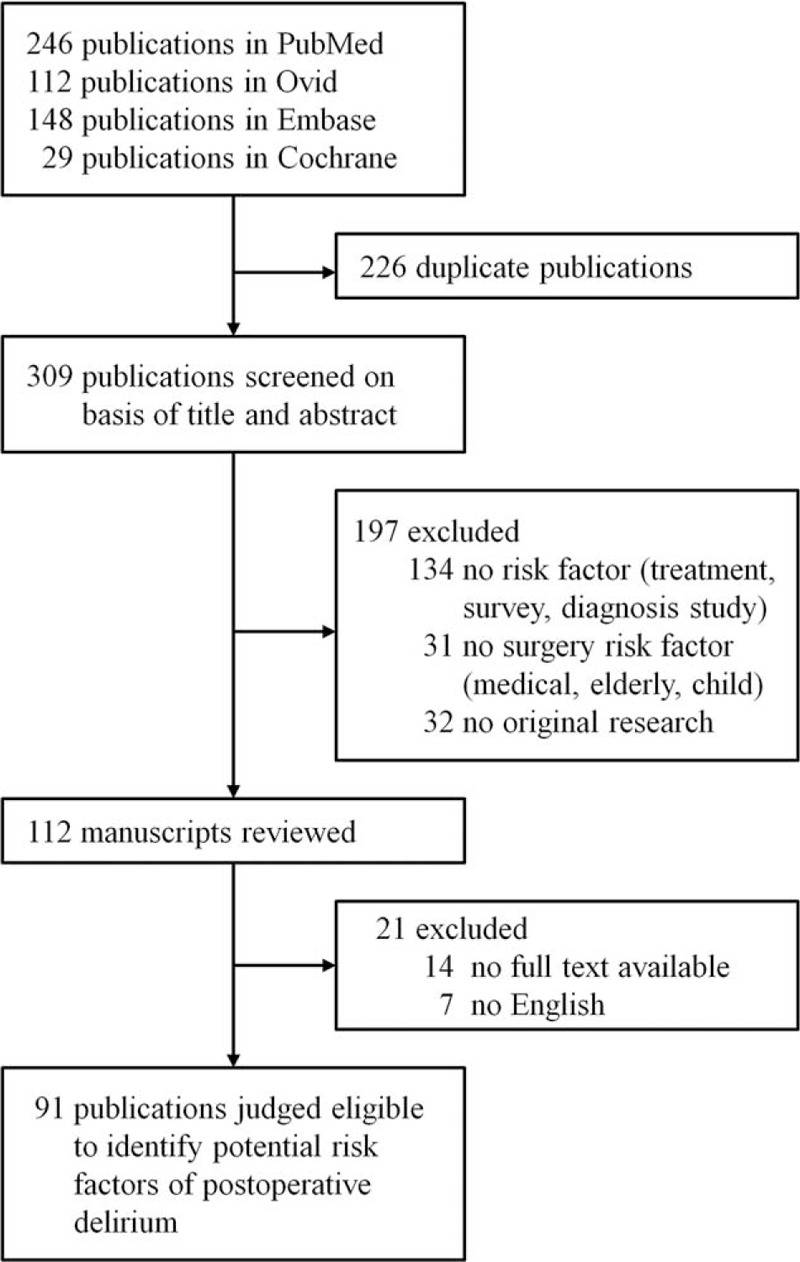
Risk factor selection process.

We conducted a comprehensive review of the literature to identify potential risk factors for postoperative delirium. The review was conducted using PubMed (www.ncbi.nlm.nih.gov/pubmed), Ovid (gateway.ovid.com), EMBASE (www.elsevier.com), and Cochrane (www.cochranelibrary.com) in February 2013. The keywords were as follows “(delirium OR confusion) AND (risk factors OR factors) AND (surgery OR operation OR postoperative)” (Figure 1). Language was limited to English and publication years to 1990 to 2012 because the Confusion Assessment Method (CAM) was used since 1990.^18^

### Diagnosis of Delirium

Trained nurses assessed patients using a nursing delirium screening checklist (Nu-DESC)^19^ at each shift and whenever patients showed any changes in mentality up to postoperative day 4. If a patient was positive in this screening, a physician examined the patient and diagnosed postoperative delirium using CAM. If a patient met the delirium criteria immediately postoperatively, examination was performed again 1 to 2 hours later to distinguish true delirium from the residual effects of anesthesia.

### Development of the Delphi Score

Patient recruitment started in June 2013 and was completed in January 2014. The eligible patients were as follows: patients of either sex above the age of 60 years, patients admitted with an expected duration of hospital stay of at least 3 days after major general surgery (gastrointestinal, hepato-biliary-pancreatic, colorectal, vascular, or trauma surgery). Exclusion criteria were as follows: patients unable to perform cognitive or psychometric tests for any reason, patients who had cognitive dysfunction or scored less than 24 in mini-mental status examination, patients who showed delirium on admission, and patients who were treated with mechanical ventilation under sedation.

After informed consent was obtained, data were collected from 561 patients. We collected demographic variables and information on 48 potential risk factors identified by reviewing previous studies (Table 1 ). Clinical data were collected by clinicians and nursing staff electronically within 24 hours after surgery. Intraoperative parameters were documented on the basis of anesthesia records. The American Society of Anesthesiologists (ASA) score was calculated by anesthesiologists. The pain score was recorded three times a day routinely and when the patient complained of pain during admission after surgery. Nutritional status was assessed by a nutritionist within 24 hours after admission.

**TABLE 1:**
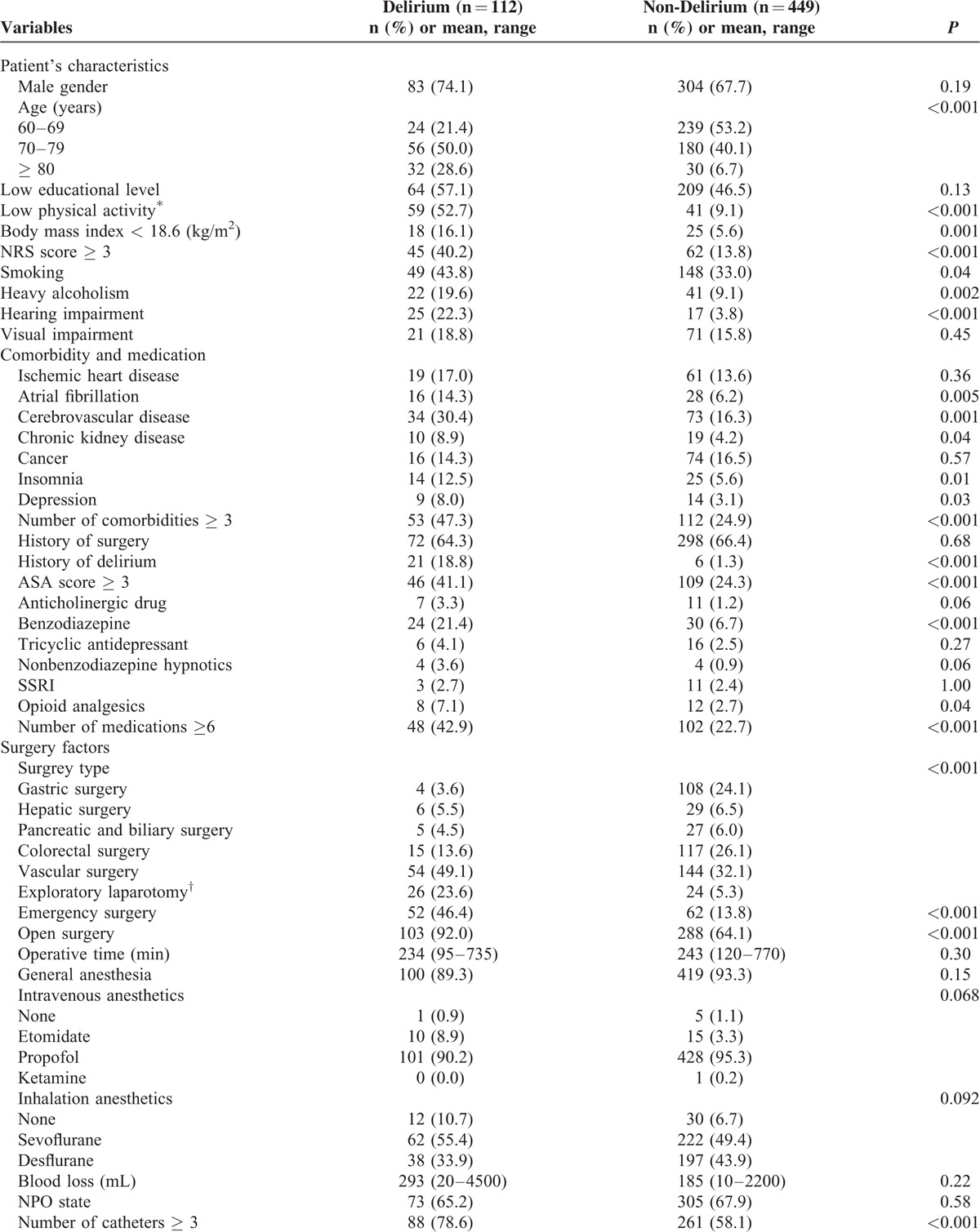
Comparison Between Delirium Patients and Non-Delirium Patients for the 48 Potential Risk Factors Identified by Previous Studies

**TABLE 1 (Continued):**
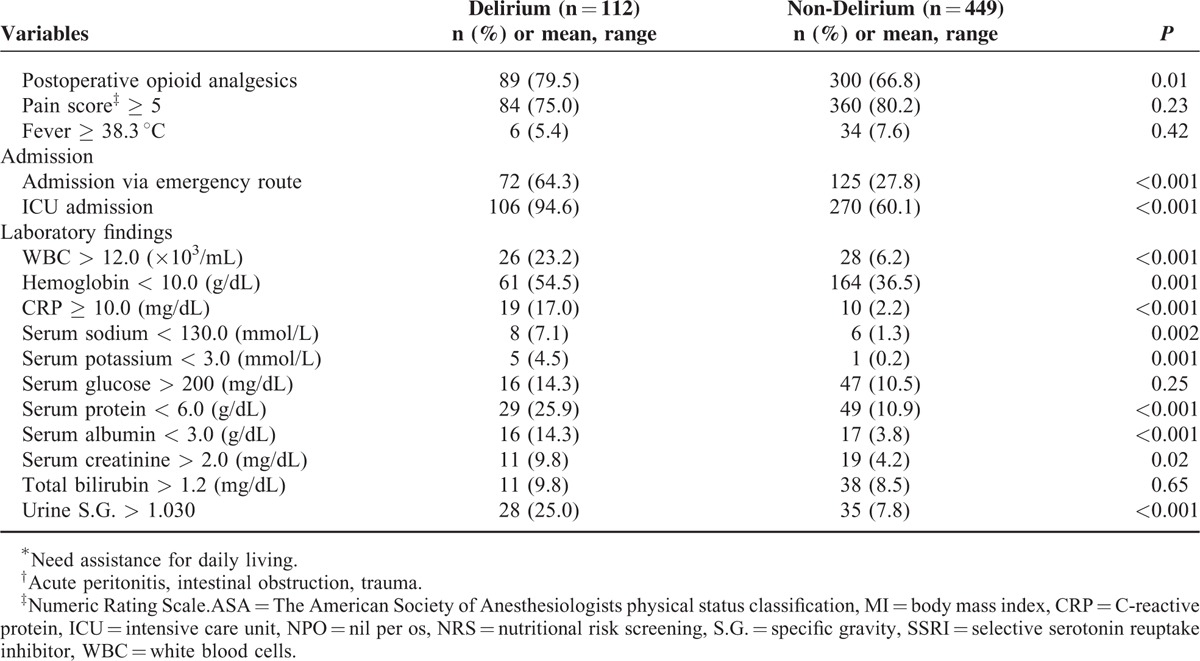
Comparison Between Delirium Patients and Non-Delirium Patients for the 48 Potential Risk Factors Identified by Previous Studies

### Validation of the Delphi Score

To validate the Delphi score, another cohort of patients was recruited from February to October 2014. After informed consent was obtained, data were collected from 553 patients at the same hospital. Inclusion and exclusion criteria were the same as for the development cohort. We assessed the sensitivity, specificity, positive predictive value (PPV), and negative predictive value (NPV) of the Delphi score.

### Statistical Analyses

**FIGURE 2:**
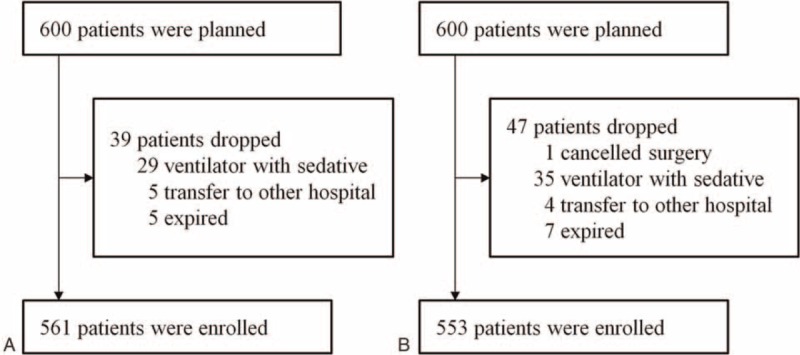
Patient enrollment in the development study (A) and validation study (B).

Sample sizes for the development and validation of the Delphi scoring system were determined as 10 patients per risk factor according to Nunnally's rule.^20^ As 48 risk factors were identified, at least 480 patients were needed. We expected a drop-out rate of 20% and planned to enroll 600 patients in each part of the study. Of the 600 eligible patients, 39 patients were dropped and 561 patients (93.5%) were enrolled in the development study (Figure 2A) and 47 patients were dropped and 553 patients (92.2%) were enrolled in the validation study (Figure 2B). All variables were collected from all enrolled patients.

Statistical analysis was performed using SPSS 20.0 (IBM SPSS Inc., Chicago, IL). For descriptive statistics, continuous variables were expressed as mean ± SD. Categorical data were expressed as number and percentage. For two-group comparisons, *t*-test or Mann-Whitney *U* test was used as appropriate. Categorical data were compared using Chi-square test or Fisher's exact test. All reported *P* values are two sided, with values less than 0.05 taken to be significant.

We used a backward stepwise logistic regression model to develop the Delphi score by assessing the association between each potential risk factor and the presence or absence of delirium. To calculate the Delphi score, regression coefficients for significant independent predictors were rounded to the nearest whole integer.

The area under the curve (AUC) was calculated using receiver operating characteristic (ROC) curve analysis. Using the Youden index, we chose the optimal cut-off point as having the highest sensitivity and specificity to discriminate between high and low probability of postoperative delirium.

In the validation study, we calculated the Delphi score of each patient in the validation cohort and calculated an AUC based on the new ROC. Finally, sensitivity, specificity, PPV, and NPV were calculated to examine how well the model performed for the prediction of delirium.

The study protocol was approved by the ethical committee of Dong San Medical Center Hospital (IRB No. 2013-05-29-001). The study was performed according to the Declaration of Helsinki.

## RESULTS

The overall prevalence of delirium in the development cohort was 20.0% (112/561). Delirium developed in 50.0% of the patients on the day of operation, in 33.9% on postoperative day 1, in 8.9% on day 2, in 5.4% on day 3, and in 1.8% on day 4. The mean duration of delirium was 3.2 ± 2.5 days.

### Possible Risk Factors for Postoperative Delirium in Bivariate Analysis

In total, 34 factors were identified as possible risk factors in bivariate analysis. Of those, predisposing factors were age, smoking, heavy alcoholism, low physical activity, low body mass index (<18.6 kg/m^2^), low nutritional risk screening score, hearing impairment, atrial fibrillation, history of cerebrovascular accident, chronic kidney disease, sleep disorders, depression, multiple comorbidities, history of delirium, high ASA score, benzodiazepine medication, neuroleptic medication, opioid analgesics, and multiple medications. Precipitating factors were emergency surgery, open surgery, multiple indwelling catheters, postoperative opioid analgesics, intensive care unit (ICU) admission, and admission to emergency room. Analysis of preoperative laboratory test results showed that high levels of C-reactive protein, leukocytosis, increased urine specific gravity, low hemoglobin, hyponatremia, hypokalemia, low serum protein, and low serum albumin were significant risk factors for postoperative delirium (Table 1 ).

### Development of the Delphi Score

A prediction model was derived from multiple logistic regression using significant risk factors from bivariate analysis. Age, low physical activity, hearing impairment, heavy alcoholism, history of prior delirium, ICU admission, emergency surgery, open surgery, and increased preoperative C-reactive protein were independent predictors of postoperative delirium (Table 2). We developed Delphi score from the above independent predictors and score range was 0 to 15 (Table 3).

**TABLE 2:**
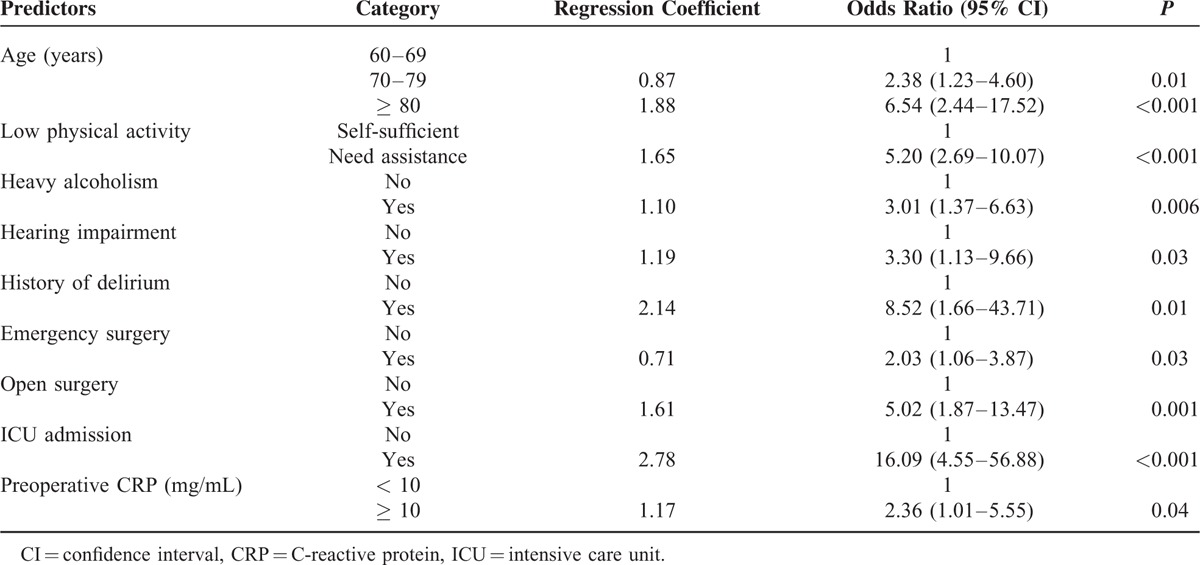
Independent Predictors of Postoperative Delirium Identified by Logistic Regression Analysis

**TABLE 3:**
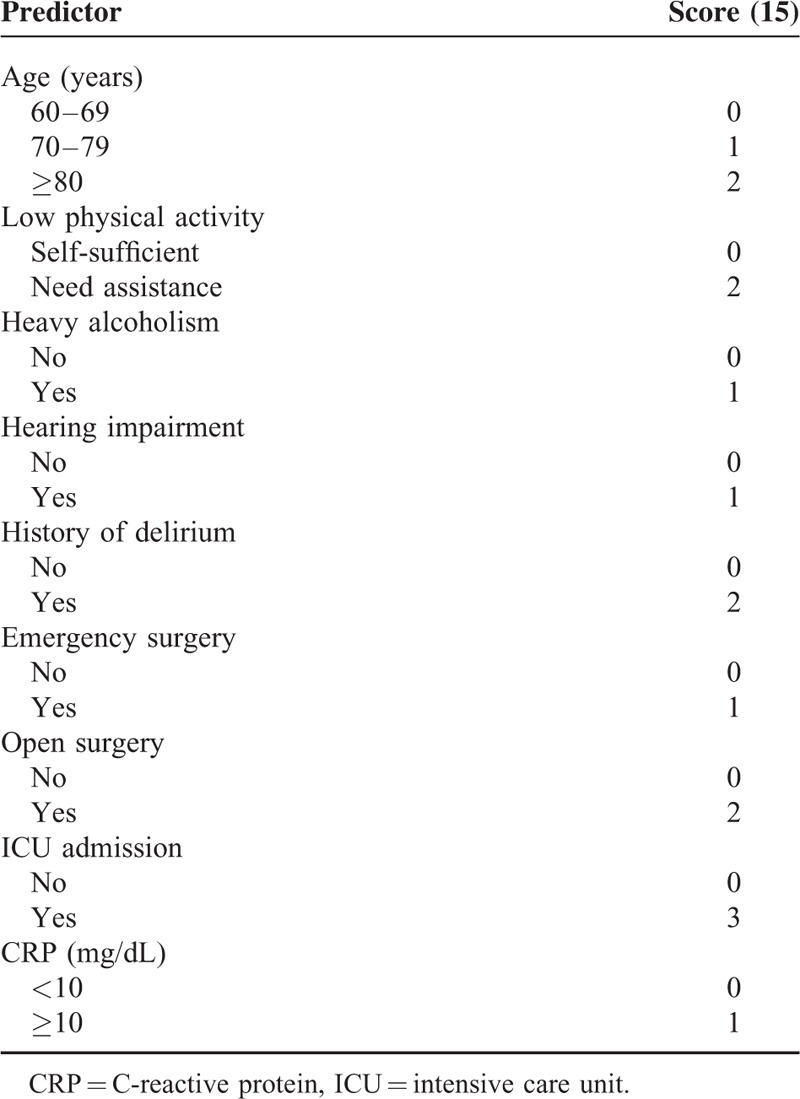
Development of the Delirium Prediction Score

**FIGURE 3:**
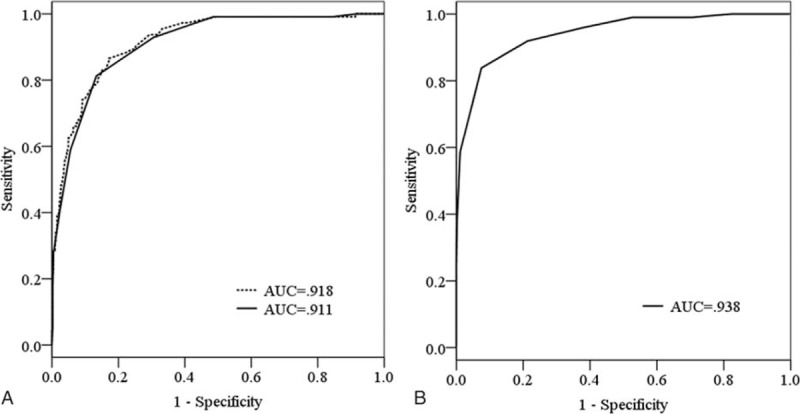
Receiver operating characteristic (ROC) curves and calculated area under the curves (AUC). (A) Development study of the Delphi score (dotted line, ROC curve of the logistic regression model; solid line, ROC curve of the Delphi score). (B) Validation study of the Delphi score. Delphi score is useful to distinguish patients with high risk of postoperative delirium from those with low risk. The cut-off value of the Delphi score corresponding to the optimal trade-off between sensitivity and specificity is 6.5. AUC = area under the curves; ROC = receiver operating characteristic.

We generated a ROC curve with predicted probabilities from the logistic regression model; the AUC was 0.918 (95% CI: 0.89–0.95) (Figure 3A). The maximum score was 15 (Table 3). A new AUC was estimated based on the Delphi score to compare it with the original AUC. The new AUC was 0.911 (95% CI: 0.88–0.94), which was similar to the original AUC (Figure 3A). The optimal cut-off point to discriminate between high and low probability of postoperative delirium was 6.5. If the Delphi score was 7 or more, the patient was classified as having a high risk of postoperative delirium, and vice versa.

### Validation of the Delphi Score

We validated the developed Delphi score prospectively. No statistical differences were found between the development and validation cohorts in terms of the distribution of the 9 predictors of postoperative delirium (Table 4). The calculated AUC based on the Delphi score was 0.938 (95% Cl: 0.91–0.97) (Figure 3B).

**TABLE 4:**
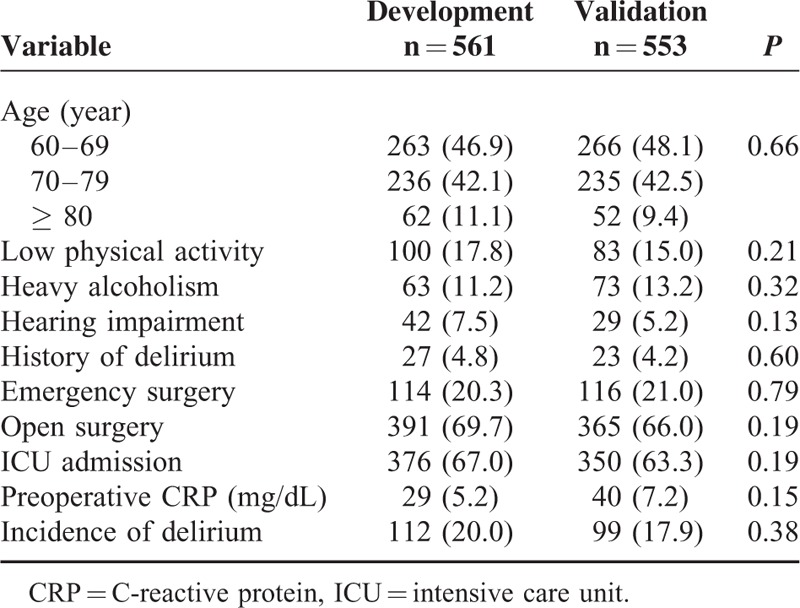
Homogeneity of General Characteristics

We divided the validation group into low- and high-risk groups with the Delphi scores of 0 to 6 and 7 to 15, respectively. The sensitivity of the delirium prediction model was 80.8%, specificity 92.5%, positive predictive value 70.2%, and negative predictive value 95.7% (Table 5).

**TABLE 5:**
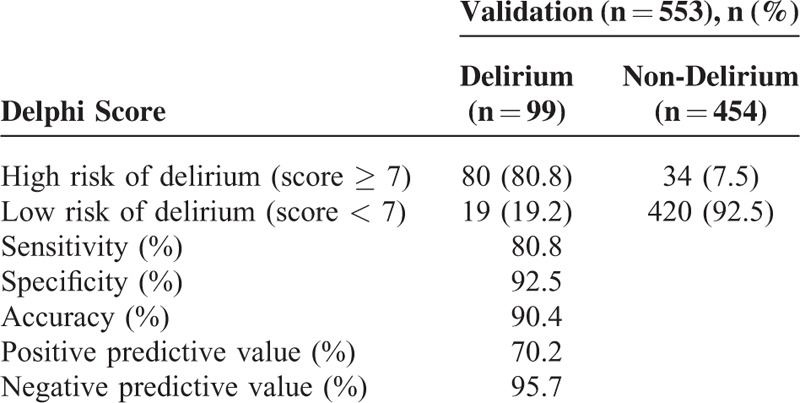
Validation of the Delirium Prediction Score

## DISCUSSION

We have developed a practical DELirium Prediction based on Hospital Information (Delphi) score. We found that prognostic information was contained in 9 predictors: age, low physical activity, hearing impairment, heavy alcoholism, history of prior delirium, ICU admission, emergency surgery, open surgery, and increased preoperative CRP. If a patient had a Delphi score 7 or more, the probability of delirium development was 80.8%.

Studies of pharmacological approaches to delirium prevention did not have convincing and reproducible evidence of effectiveness yet.^15,21^ Nonpharmacological prevention is widely accepted as an effective strategy for delirium in older hospital patients. Marcantonio et al suggested that proactive intervention reduces delirium by over 1/3 and severe delirium by over 1/2.^22^ Recent guidelines recommended delirium prevention activity in persons at risk.^23^ Since medical resources are limited, it is important to determine for whom intervention is required to prevent delirium, and this can be achieved by using the Delphi score.

This study was started by reviewing the published literature to search for potential risk factors for postoperative delirium because we thought that the delirium prediction score would be more reliable if it includes already identified risk factors. All of the 9 identified predictors were described in the prior literature as risk factors for postoperative delirium. Other factors evaluated in this study had no added value for the prediction of postoperative delirium when the nine predictors were taken into account, which, however, does not mean that these factors are not important.

This study has several limitations. First, as it did not include orthopedic, obstetric, or neurosurgery patients, our results cannot be easily generalized to all surgical patients. The departments that treat the above groups of patients had a similar increase in the numbers of elderly patients, and we need to validate the Delphi score for patients from these departments. Second, the Delphi score has been validated internally but not yet externally in another population. Third, we used a cut-off value to discriminate between high- and low-risk patients. However, the risk of postoperative delirium is actually not dichotomous but continuous. Last, there could be a potential misdiagnosis for the hypoactive delirium without psychomotor behavioral changes, because their symptoms are passive and might be delayed.

An important strength of this study is that it was a prospective study that enrolled general surgery patients and that the predictors in our model are well-defined and easily measured clinical variables. Although this model has not yet been validated externally in other hospitals, the Delphi score showed a high predictive value in our internal validation study.

## CONCLUSIONS

Our proposed Delphi score, which is based on easily available patients’ and clinical information, could help surgeons and nursing staff to predict the development of delirium in postoperative patients and make possible targeted delirium prevention in the high-risk group. Further studies are needed to validate this score and to assess its clinical usefulness.
